# Supplementary material for: Sex and Diet-Related Disparities in Low Handgrip Strength among Young and Middle-Aged Koreans: Findings Based on the Korea National Health and Nutrition Examination Survey (KNHANES) from 2014 to 2017
Source: Nutrients. 2022 Sep 15;14(18):3816. doi: 10.3390/nu14183816 (PMC9504322; doi:10.3390/nu14183816)
Supplement: Supplementary file 1 [file nutrients-14-03816-s001.zip › nutrients-1873993-supplementary.pdf]

Supplementary Table S1. Factor loading matrix for major dietary pattern of the men.

Food group	Factor1<br>Westernized Korean	Factor2<br>Convenience	Factor3<br>Traditional Korean
Seasonings	0.65	0.10	0.01
Oils	0.62	0.29	0.04
Meats	0.49	-0.03	-0.24
Sugar and sweets	0.44	0.21	0.13
Fish and shellfish	0.40	-0.17	0.12
Non-sugar beverages	0.33	0.05	0.06
Functional foods	0.02	-0.02	0.01
Flour and bread	-0.04	0.57	-0.06
Milk and dairy products	-0.06	0.50	0.07
Soda	0.09	0.43	-0.28
Processed food	0.09	0.32	-0.05
Eggs	0.10	0.27	0.19
Processed meats	0.02	0.22	0.02
SSBs	0.00	-0.06	0.02
Alcohol	0.41	-0.41	-0.31
White rice	0.01	-0.31	0.52
Legumes	0.05	-0.09	0.49
Whole grains	-0.12	0.09	0.47
Fruit and vegetable	0.27	0.13	0.43
Kimchi	-0.03	-0.29	0.41
Seaweeds	-0.03	-0.05	0.36
Nuts	0.06	0.08	0.27
Mushrooms	0.15	0.09	0.21
Potatoes	0.07	0.12	0.12
Noodles	-0.06	0.15	-0.31
**Kaiser's Measure of Sampling Adequacy: 0.576**			
**Bartlett'chi-square: 6424.505 (p value: <.001)**			

SSBs, sugar sweetened beverages. Numbers in bold indicate that the factor loading score is greater than |0.25| in men.

Supplementary Table S2. Factor loading matrix for major dietary pattern of the women.

Food group	Factor1<br>Westernized Korean	Factor2<br>Traditional Korean	Factor3<br>Healthy
Oils	0.68	-0.02	-0.14
Seasonings	0.54	-0.01	-0.20
Sugar and sweets	0.48	-0.12	0.11
Non-sugar beverages	0.37	-0.15	0.14
Fish and shellfish	0.30	0.20	-0.07
Eggs	0.26	0.07	0.09
Mushrooms	0.23	0.07	0.02
Processed food	0.16	-0.10	-0.11
White rice	0.00	0.68	-0.17
Kimchi	-0.01	0.59	-0.09
Legumes	0.13	0.39	0.20
Seaweeds	0.08	0.34	0.14
Functional foods	0.06	0.07	0.06
Noodles	0.00	-0.28	-0.05
Soda	0.22	-0.30	-0.29
Milk and dairy products	0.16	-0.32	0.30
Flour and bread	0.22	-0.33	0.04
Nuts	0.20	0.05	0.44
Fruit and vegetable	0.34	0.24	0.39
Whole grains	0.04	0.31	0.38
Potatoes	0.07	0.00	0.26
Processed meats	0.08	-0.03	-0.17
SSBs	0.01	0.08	-0.20
Alcohol	0.12	-0.02	-0.43
Meats	0.37	-0.02	-0.45
**Kaiser's Measure of Sampling Adequacy: 0.575**			
**Bartlett'chi-square: 6650.418 (p value: <.001)**			

SSBs, sugar sweetened beverages. Numbers in bold indicate that the factor loading score is greater than |0.30| in women.
